# Exploring Near-Peer Teaching for Examination Preparation Among Undergraduate Medical Students: Perceptions of Peer Tutors and Tutees

**DOI:** 10.7759/cureus.87018

**Published:** 2025-06-30

**Authors:** Aarati B Pokale, Mandar Karmarkar

**Affiliations:** 1 Community Medicine, Bharati Vidyapeeth (Deemed University) Medical College, Pune, IND; 2 Forensic Medicine, Bharati Vidyapeeth (Deemed University) Medical College, Pune, IND

**Keywords:** exam, mbbs, medical undergraduates, near-peer teaching, npt

## Abstract

Background: Near-peer teaching (NPT), where students take on the role of educators for peers, is widely used in medical education to enhance the learning experience. We sought to explore the perspectives of near-peer tutors and the advantages of NPT among tutors and tutees in the context of exam preparation.

Implementation: We conducted NPT sessions for examination-oriented revision.

Results: The feedback included rating the session and open-ended questions for scope and for free-text comments. Likert scale analysis revealed that almost 50% of tutees strongly agreed that tutors have a strong knowledge base, clarity of concepts, and an approachable attitude, and provided valuable resource materials. Free text comments were categorized into common themes: empathy and relatability, studying tips, drafting answers, tailored guidance, exam-specific approach, and precise revision.

Conclusion: Based on the feedback of tutees and tutors, initiating NPT in medical colleges for exam preparation will go a long way in benefiting the students.

## Introduction

Peer teaching, where students take on the role of educators for their peers, is a widely used method in medical education to enhance the learning experience [1]. Near-peer teaching (NPT) is a specific approach where a senior student, typically one or more years ahead, teaches a junior student [2-4]. NPT is characterized by a formal relationship in which a more qualified student provides guidance and support to a less qualified student [2,5]. NPT is rooted in the idea of social and cognitive congruence, which suggests that students find instruction from those closer to their training stage more relatable and accessible than that of senior clinicians or faculty members [1,6,7]. This congruence helps create a supportive and encouraging environment where students feel more comfortable asking questions, discussing their difficulties, and learning from the specific experiences of their near-peers [1,8,9]. Near-peer tutors can recall their examination experiences and guide their tutees on the specifics of the examination and preparation required [5].

NPT is seen as a way to support medical students' learning while developing the teaching skills of senior students [2,5,8]. Studies have shown that NPT helps tutors and tutees become better physicians [1]. Many medical schools have integrated NPT into their curricula to enhance student learning [5].

Near-peer tutors share a common background with their tutees and thus easily create an atmosphere of trust and empathy. They understand the challenges that tutees may face and offer advice and support based on their own experiences [2,5,8-10].

Studies have delved into the various dimensions of NPT within medical education. Some have focused on the benefits for both tutors and tutees, emphasizing enhancements in knowledge, skills, and confidence [2,5,7,10,11]. Others have highlighted the effectiveness of NPT across diverse settings, anatomy labs, clinical skills training, and virtual learning environments in medical schools [2,5,9]. This study aimed to analyze the perspectives of tutors and tutees, as well as the advantages of NPT, in the context of exam preparation, a critical component of medical education.

## Materials and methods

The NPT program for exam preparation was conducted for undergraduate medical students at Bharati Vidyapeeth (Deemed University) Medical College, a private medical college in Pune, Maharashtra, India.

Preparatory phase

We planned the NPT sessions as an examination-oriented revision. We circulated an online form in the class WhatsApp (Meta Platforms, Inc., Menlo Park, CA) groups of first- and second-year Bachelor of Medicine, Bachelor of Surgery (MBBS) students. The form outlined the concept of NPT while seeking suggestions on subjects and topics for which the students desired guidance for exam preparation. It also stated that participation in the program was voluntary.

Selection of peer tutors

A meeting was conducted with third-year MBBS students, as they were the near-peer tutors. NPT was explained to them in detail, along with examples of its applications in medical education, its benefits, and its challenges. The choice of subject and topics was left to the tutor. A strong academic record and comprehensive knowledge in the subject they wished to revise were mandatory. To ensure a successful and impactful peer teaching program, the following criteria were emphasized: 1. Effective communication skills; 2. Commitment to the program, as they had to invest time and effort to prepare and conduct the sessions; 3. Time management to manage their studies and peer-teaching responsibilities equally effectively; 4. Technical and resource development skills were desirable. Proficiency in using educational tools and platforms and ability to develop useful learning resources (e.g., handouts, presentations, videos, etc.); 5. Any previous tutoring experience was a bonus. Of the 20 students from the third year MBBS who expressed interest in tutoring, 14 were shortlisted based on the above criteria. 

Implementation

Micro-teaching sessions (10-15 minutes in duration) were conducted for the 14 peer tutors to assess their communication skills and ability to explain concepts.

The peer tutors were instructed to refer to standard textbooks while preparing content. It is a routine practice of the institute to share learning resources prepared by faculty on the institutional Learning Management System (LMS) for students. All peer tutors stated that they planned to refer to these resources. 

The sessions were conducted for two to 2.5 hours, once or twice a week after regular college hours for three months. The schedule was mutually adjusted to suit both tutors' and tutees' requirements. The sessions were conducted on the online platform Microsoft Teams (Microsoft Corp., Redmond, WA), as all students had access to it through their official institutional email accounts. 

At the end of each session, the peer tutors uploaded the learning resources (PowerPoint presentations (Microsoft Corp.), videos, etc.) on the LMS for students to access in the future, if required.

Feedback

At the end of each session, an anonymous online feedback form was shared with tutees. The questionnaire was pilot tested and validated by experts. Feedback on the quality of the discussion and learning resource material, pace of discussion, interaction, and checking for understanding was sought (Appendix A). This feedback was shared with the tutors to ensure prompt improvement, if required.

A five-point Likert scale was used to rate the sessions (quality, duration, effective use of audio-visual aids, content created) and the tutors (preparedness, interaction, checking for understanding) [5]. An open-ended question sought detailed inputs about the sessions. 

Statistical analysis

The data were directly obtained from a Microsoft Excel file (Microsoft Corp.) generated from the online feedback forms. Frequencies and percentages were calculated in Microsoft Excel. 

## Results

A total of 888 feedback forms were received for the 15 sessions conducted. Though 100 students initially enrolled for the program, attendance varied with each session. The Likert scale ratings revealed a positive response from over 90% of the students (Table 1). 

**Table 1:** Feedback from near peer tutees on a five-point Likert scale (n=888) PT: peer tutor

Question	Overall responses (%)
1. The PT revised concepts clearly and effectively	
Strongly disagree	0 (0)
Disagree	0 (0)
Neutral	0 (0)
Agree	375 (42.2)
Strongly agree	513 (57.8)
2. The PT demonstrated a good understanding of the subject matter	
Strongly disagree	0 (0)
Disagree	0 (0)
Neutral	19 (2.1)
Agree	423 (47.6)
Strongly agree	446 (50.2)
3. The PT encouraged active participation and engagement from students	
Strongly disagree	0 (0)
Disagree	0 (0)
Neutral	28 (3.2)
Agree	381 (42.9)
Strongly agree	479 (53.9)
4. The PT was approachable and responsive to questions	
Strongly disagree	0 (0)
Disagree	0 (0)
Neutral	27 (3)
Agree	337 (38)
Strongly agree	524 (59)
5. The PT used appropriate teaching methods and resources to enhance sessions	
Strongly disagree	0 (0)
Disagree	0 (0)
Neutral	21(2.4)
Agree	323 (36.4)
Strongly agree	544 (61.3)
6. Overall, the sessions were valuable for my exam preparation	
Strongly disagree	0 (0)
Disagree	0 (0)
Neutral	17 (1.9)
Agree	361 (40.7)
Strongly agree	510 (57.4)
7. Pace of teaching	
Adequate	830 (93.5)
Fast	52 (5.9)
Slow	6 (0.7)

For feedback on the sessions, qualitative themes and representative quotes of tutees and tutors are shown in Tables 2, 3, respectively. 

**Table 2:** Qualitative themes and common quotes identified from tutees when asked about the experience of sessions conducted by the peer tutors for exam preparation (n=888)

Common themes identified from tutees' responses	Direct quotes from tutee responses
Empathy and relatability	''Seniors, having been in similar positions, provided a relatable perspective and addressed common difficulties.''
	''This helped as it came from a student’s perspective''
	''Along with that, as these seniors were once in our place, they know the areas of difficulties along with the type of questions asked, making this session super interesting!''
Studying tips	''Strategies to remember and reproduce information during exams, including mnemonics and memory aids.''
	''Very helpful in quick revision and tips regarding exams''
	''Important topics, frequently asked questions, and concepts to focus on for exams''
Drafting answers	'Guidance on how to structure answers & use concise language''
	''I was looking forward to guidance on viva questions, emergency drugs viva, etc. The session was on point.''
	''The model answers which are uploaded regarding the ………….. and give reasons are really helpful, since I personally feel it is difficult to write the answers in a correct manner.''
	''Structured format, emphasis on a question-oriented teaching style and structured answer presentation.''
	''Discussion of answers on how to write in exams and what topic should be focused on more''
Tailored guidance	''Insights into specific problem areas, including writing pharmacotherapy answers''
	''Tackling viva questions and handling emergency topics.''
	''They were telling us the type of questions asked, giving us a different perspective on how to answer the question''
	''Highlighting critical areas for studying while preparing for exams''
Exam-specific approach and precise revision	''Addressed how to prioritise and present content under exam conditions''
	''Self-made mnemonics: creation of easy-to-remember mnemonics tailored to exam requirements''
	''It was very helpful to brush up on concepts and have a quick recap, and the types of questions asked in exams''
	''Well taken care of the past papers discussion''
	''How to answer questions in a viva was told, important points''
General comments	''Overall, it was a very well-organised session and it was a great in-depth revision for the already studied topics''
	''It helped revise concepts thoroughly''
	''Nice method and ways of teaching (via live video+ cut outs + handmade notes and mnemonics)''
	''A nice revision of what had been taught by faculty, as it was taught by the teachers earlier, it felt easy to revise''

**Table 3:** Qualitative themes and common quotes identified from near-peer tutors when asked about their experience of conducting sessions for near peers for exam preparation (n=14)

Themes identified from free text analysis of feedback from tutors	Quotes of tutors
1. Self-learning	''Self-revision & improved understanding of subject/topic while preparing to teach''
	''I think we can learn better when we teach, or explain to our friends any topic, the concept stays with us for long.''
	''Improve my own understanding ''
	''Teaching a topic or subject increases the knowledge of the subject while benefiting the student too, and I also hope to be able to tell my juniors the things to focus on before the exams so they know what to prioritise. ''
	''If I’m able to teach someone, I’ll be able to review and revise some of my topics.''
2. Soft skills development	''Honing of communication skills''
	''Development of interpersonal skills like empathy and patience - ability to empathise with students’ difficulties and be patient in explaining concepts multiple times if necessary.''
3. Bonding	''Experience sharing which will benefit tutees''
	''Strengthening student relationships & fostering a collaborative learning culture''
	''Genuine Interest in teaching: I have a passion for teaching and helping others.''
	''Being approachable so that peers feel comfortable asking questions.''
	''It’s a chance to help our juniors with whatever difficulties they have''
	''Senior-junior interaction in a more professional environment.''
	''I could share my experiences with them, I could guide them to not make the same mistakes I made while studying, and it will help me revise previous topics.''
	''It’ll help erase the stigma of being unable to ask your seniors for help.''
	''Just wanted to try to make remembering a little easier and maybe give them ways to remember. Also, can tell them various questions that are commonly asked during viva or theory papers.''

## Discussion

Peer teaching, particularly NPT, is a widely used educational method in undergraduate medical education [1-5]. Justifying the role of NPT in medical colleges in India involves highlighting its benefits and relevance within the context of medical education and healthcare delivery. Here are the ABCDs of NPT for consideration: A for "Aligning with global trends": NPT is recognized as a valuable educational strategy in medical education worldwide. Including this in medical colleges in India is in line with global educational best practices [1,12,13,14]; A for "Accreditation standards": Accrediting bodies often value innovative teaching methods. Incorporating near-peer teaching into Indian medical education programs can help medical colleges meet accreditation standards. B for "Better exam performance": Some studies suggest that peer teaching programs may enhance student-learners' performance on examinations [9,11,15,16]. C for "Confidence": NPT helps junior students build confidence in their knowledge and skills and approach to examinations, as they learn from peers who have recently undergone similar experiences. Similarly, donning the hat of peer tutor instills confidence in the peer tutor. Also, teaching reinforces the teacher's learning, leading to better knowledge retention and deeper understanding of the content [1,3,5,6,14,16]. C for "Congruence": The rationale behind NPT is often supported by the concepts of social and cognitive congruence [1-4,6-22]. D for "Development of pedagogical and soft skills": NPT aids the development of essential soft skills, such as communication, teamwork, and leadership, in students. Senior students develop valuable teaching and communication skills, beneficial for future careers [1-5,7-9,12-15,18, 21,22].

Post program, we offered a combination of incentives to enhance the appeal of peer tutoring in the future and ensured that peer tutors felt valued and motivated. From the start of the NPT program, we assigned leadership roles to the peer tutors, and they planned and executed the sessions. Since conducting examination revision sessions was the brainchild of the students, they felt intrinsically motivated and hence engaged in all decision-making processes. 

We awarded certificates of participation and appreciation to the 14 peer tutors in an official institutional ceremony. Such public accolades serve to motivate the future generations of near-peer tutors. 

Some studies advocate financial incentives, academic credit points, access to paid courses or learning material, etc., to motivate peer tutors [10,18].

Though there have been instances of peer teaching being viewed negatively, especially with regard to the proficiency of peer teachers, we received positive feedback [2,11]. As stated by some students, it could be because this was like a revision of what was taught in class by the faculty.

NPT is not without its share of challenges, with ensuring adequately trained peer teachers delivering accurate and appropriate content being the oft-cited one. Also, resource constraints and time management could be potential deterrents [1-3, 20]. Using an online teaching mode like we did and engaging students after regular college hours with a pre-designed schedule to coincide with the examination preparatory period will help overcome these challenges.

Limitations of the study

We conducted the NPT program for examination preparation as a one-time program a few months before just one examination. Hence, we did not analyze the examination results to assess the impact of the activity. Regular scheduling of similar sessions before each examination and analysis of marks thereafter will shed more light on its benefits. Comparing outcomes in terms of examination results of near-peer tutored students with a control group will give a more scientific analysis. Also, this program was implemented at a single center. To test its robustness, these should be conducted across multiple medical colleges for students from all years. 

## Conclusions

This study sought perceptions of both parties involved in NPT for examination preparation. The positive feedback goes a long way in recommending this activity and encouraging senior students to volunteer for it. The online format for conducting it creates a comfortable learning environment. Due to the cognitive and social congruence between the two types of students involved, this is especially beneficial in preparation for examinations.

We recommend that a longitudinal study exploring the impact of NPT for examination preparation on results (formative as well as summative) will be of greater value. An experimental study design with long-term comparison of examination results and knowledge retention of near-peer-tutored students with a control group is recommended. Also, conducting it across multiple medical colleges for students from all years is recommended to test its benefits. 

Considering the current classroom scenarios with the Competency-Based Medical Education (CBME) model and the focus on blended learning, self-directed learning, etc., this model of NPT for examination preparation or revision seems worth adopting.

## Appendices

Appendix A 

**Table 4:** Questionnaire PT: peer tutor

	Questionnaire		
A	Select year (I MMBS or II MBBS)		
B	Gender	Male	
		Female	
		Prefer not to say	
C	Mention the subject for which you are giving feedback		
D	Mention the topic of the subject for which you are giving feedback		
E	Mention the name of the near-peer tutor whose session you are giving feedback on		
F	Rate the sessions on the Likert Scale: a= Strongly Disagree, e= Strongly Agree		
	1. The near-peer tutor revised concepts clearly and effectively		
	a - Strongly Disagree		
	b - Disagree		
	c - Neutral		
	d - Agree		
	e- Strongly Agree		
	2. The PT demonstrated a good understanding of the subject matter		
	a - Strongly Disagree		
	b - Disagree		
	c - Neutral		
	d - Agree		
	e- Strongly Agree		
	3. The PT encouraged active participation and engagement from students		
	a - Strongly Disagree		
	b - Disagree		
	c - Neutral		
	d - Agree		
	e- Strongly Agree		
	4. The PT was approachable and responsive to questions		
	a - Strongly Disagree		
	b - Disagree		
	c - Neutral		
	d - Agree		
	e- Strongly Agree		
	5. The PT used appropriate teaching methods and resources to enhance sessions		
	a - Strongly Disagree		
	b - Disagree		
	c - Neutral		
	d - Agree		
	e- Strongly Agree		
	6. Overall, the sessions were valuable for my exam preparation		
	a - Strongly Disagree		
	b - Disagree		
	c - Neutral		
	d - Agree		
	e- Strongly Agree		
	7. Pace of teaching		
	Adequate		
	Fast		
	Slow		
	Numerical rating a= strongly disagree, e= strongly agree		
G	Give your constructive comments on the session		
H	List of topics for future discussion		

## References

[1] Tanveer MA, Mildestvedt T, Skjærseth IG (2023). Peer teaching in undergraduate medical education: what are the learning outputs for the student-teachers? A systematic review. Adv Med Educ Pract.

[2] Khapre M, Deol R, Sharma A, Badyal D (2021). Near-peer tutor: a solution for quality medical education in faculty constraint setting. Cureus.

[3] de Menezes S, Premnath D (2016). Near-peer education: a novel teaching program. Int J Med Educ.

[4] Bowyer ER, Shaw SC (2021). Informal near-peer teaching in medical education: a scoping review. Educ Health (Abingdon).

[5] Baskaran R, Mukhopadhyay S, Ganesananthan S (2023). Enhancing medical students` confidence and performance in integrated structured clinical examinations (ISCE) through a novel near-peer, mixed model approach during the COVID-19 pandemic. BMC Med Educ.

[6] Knobloch AC, Ledford CJ, Wilkes S, Saperstein AK (2018). The impact of near-peer teaching on medical students’ transition to clerkships. Fam Med.

[7] Zhu MM, Brennan MT (2025). Near-peer perspectives on a voluntary peer teaching program: challenges and solutions. Med Sci Educ.

[8] Orsini E, Quaranta M, Mariani GA (2021). Near-peer teaching in human Anatomy from a tutors’ perspective: an eighteen-year-old experience at the University of Bologna. Int J Environ Res Public Health.

[9] Burgess A, Dornan T, Clarke AJ, Menezes A, Mellis C (2016). Peer tutoring in a medical school: perceptions of tutors and tutees. BMC Med Educ.

[10] Messerer DA, Kraft SF, Horneffer A, Messerer LA, Böckers TM, Böckers A (2022). What factors motivate male and female Generation Z students to become engaged as peer teachers? A mixed-method study among medical and dental students in the gross anatomy course. Anat Sci Educ.

[11] Hielscher AC, Everse S (2022). Virtual peer teaching in the gross anatomy lab: a format of peer teaching and learning during the COVID-19 pandemic. MedEdPublish (2016).

[12] Nelson AJ, Nelson SV, Linn AM, Raw LE, Kildea HB, Tonkin AL (2013). Tomorrow's educators … today? Implementing near-peer teaching for medical students. Med Teach.

[13] Chen JY, Lam TP, Hung IF, Chan ACY, Chin W-Y, See C, Tsang JPY (2023). Peer-to-peer clinical teaching by medical students in the formal curriculum. Asia Pac Scholar.

[14] Devani P, Thakker A, Shah N (2023). Near-peer-teaching revision series during the COVID-19 recovery phase: an experience from a UK medical school. Cureus.

[15] Pierce B, van de Mortel T, Allen J, Mitchell C (2024). The influence of near-peer teaching on undergraduate health professional students' self-efficacy beliefs: a systematic integrative review. Nurse Educ Today.

[16] Burgess A, McGregor D, Mellis C (2014). Medical students as peer tutors: a systematic review. BMC Med Educ.

[17] Williams B, Fowler J (2014). Can near-peer teaching improve academic performance?. Int J High Educ.

[18] Bulte C, Betts A, Garner K, Durning S (2007). Student teaching: views of student near-peer teachers and learners. Med Teach.

[19] Engels D, Haupt C, Kugelmann D, Dethleffsen K (2021). The peer teachers' perception of intrinsic motivation and rewards. Adv Physiol Educ.

[20] Loda T, Erschens R, Nikendei C, Giel K, Junne F, Zipfel S, Herrmann-Werner A (2020). A novel instrument of cognitive and social congruence within peer-assisted learning in medical training: construction of a questionnaire by factor analyses. BMC Med Educ.

[21] Zheng B, Wang Z (2022). Near-peer teaching in problem-based learning: perspectives from tutors and tutees. PLoS One.

[22] Bugaj TJ, Blohm M, Schmid C (2019). Peer-assisted learning (PAL): skills lab tutors' experiences and motivation. BMC Med Educ.

